# Dynamic optimization of stand structure in *Pinus yunnanensis* secondary forests based on deep reinforcement learning and structural prediction

**DOI:** 10.3389/fpls.2025.1610571

**Published:** 2025-10-15

**Authors:** Jian Zhao, Jianming Wang, Jiting Yin, Yuling Chen, Baoguo Wu

**Affiliations:** ^1^ School of Mathematics and Computer Science, Dali University, Dali, China; ^2^ Dali Forestry and Grassland Science Research Institute, Dali, China; ^3^ Institute of Remote Sensing and Geographic Information System, School of Earth and Space Sciences, Peking University, Beijing, China; ^4^ School of Information Science and Technology, Beijing Forestry University, Beijing, China

**Keywords:** multi-agent deep reinforcement learning, stand structure, multi-objective optimization, structure prediction, secondary forests

## Abstract

**Introduction:**

The rational structure of forest stands plays a crucial role in maintaining ecosystem functions, enhancing community stability, and ensuring sustainable management. Although progress has been made in stand structure optimization, most existing studies focus on static improvements and fail to adequately capture the dynamic nature of stand development. In addition, commonly used heuristic and traditional methods often suffer from limitations in computational efficiency and generalization ability.

**Methods:**

To address these challenges, this study explores the potential and advantages of multi-agent deep reinforcement learning in forest management, offering innovative insights and methods for achieving sustainable forest ecosystem management. Using the secondary forests of *Pinus yunnanensis* in southwest China as the research subject, we constructed an objective function and constraints based on spatial and non-spatial structure indexes. Selective harvesting and replanting were employed as optimization measures, and experiments were conducted on five circular plots to compare the performance of multi-agent deep reinforcement learning with that of multi-agent reinforcement learning. To account for the dynamic characteristics of stand structure, we further integrated structure prediction with multi-agent deep reinforcement learning for dynamic optimization across the five plots.

**Results:**

The results indicate that multi agent deep reinforcement learning consistently outperformed multi agent reinforcement learning across all plots. For the initial objective function values of each plot (0.3501, 0.3799, 0.3982, 0.3344, 0.4294), the optimized results obtained through multi agent deep reinforcement learning (0.5378, 0.5861, 0.5860, 0.5130, 0.6034) were significantly superior to the maximum objective function values achieved by multi agent reinforcement learning (0.5302, 0.5369, 0.5766, 0.5014, 0.5906). Furthermore, the dynamic optimization results incorporating structure prediction demonstrate that all plots progressively approached an ideal stand condition over multiple optimization cycles (0.5718, 0.6101, 0.6455, 0.5863, 0.6210), leading to a more balanced stand structure and improved long-term stability.

**Discussion:**

This study proposes a novel stand structure optimization method that integrates multi agent deep reinforcement learning with structure prediction, providing theoretical support and practical guidance for the sustainable management of *Pinus yunnanensis* secondary forests.

## 1 Introduction

Secondary forests often face challenges such as unstable stand structure, reduced biodiversity, increased risk of forest fires, and susceptibility to natural disturbances, including insect infestations, diseases, and wildfires (Lei et al., 2008; Ding and Zang, 2021; Zaizhi, 2001). To enhance the stability and sustainability of secondary forests, stand structure optimization has become a key technical approach in forest management and planning, providing essential support for their scientific management.

Selective harvesting is a crucial measure for optimizing stand structure and has received widespread attention (Dong et al., 2022; Dongsheng et al., 2020; Chi et al., 2019; Dong et al., 2020). By removing trees with limited growth potential and weak competitiveness, the growth environment and resource allocation of the remaining trees can be improved, thereby optimizing stand structure.

Common stand structure optimization algorithms include heuristic methods such as particle swarm optimization (PSO) (Wu et al., 2022), Monte Carlo algorithm (Haight and Travis, 1997; Boston and Bettinger, 1999), and genetic algorithms (GA) (Jianming et al., 2017; Okasha and Frangopol, 2009; Fotakis et al., 2012). PSO provides certain advantages in global search but is prone to local optima in complex problems; Monte Carlo methods explore the solution space through random sampling but often suffer from low computational efficiency; GA performs well in handling nonlinear problems but typically requires many iterations to converge. Overall, although these methods can address stand structure optimization tasks, they generally face limitations such as high computational cost, susceptibility to local optima, and insufficient solution efficiency. In our previous study, we applied deep reinforcement learning to improve the efficiency and accuracy of multi-objective stand structure optimization. By modeling tree-felling decisions as agent actions and incorporating neural networks with experience replay for stable training, this approach achieved superior optimization results compared with traditional heuristic algorithms and conventional reinforcement learning methods across multiple plots of Pinus yunnanensis secondary forests (Zhao et al., 2024).

A single selective harvesting measure can only reduce competition and adjust stand density, but it cannot restore species diversity or fill the spatial gaps created by harvesting; therefore, it is insufficient to achieve comprehensive optimization of stand structure. On this basis, replanting measures, namely the planting of native tree seedlings in appropriate locations, should be implemented to achieve overall optimization of stand structure. Common strategies for selecting replanting location include the Voronoi diagram method (Wang et al., 2019), the maximum Delaunay triangulation area method (Chunyan and Jiping, 2017), and the Kriging interpolation method (Jian et al., 2018). In general, these methods identify relatively sparse areas within the stand as potential replanting sites using different algorithms. However, they have certain limitations: on one hand, the replanting locations are relatively fixed and lack flexibility, which may lead to suboptimal replanting outcomes; on the other hand, these methods often overlook the competitive interactions between the replanting trees and neighboring trees, potentially increasing resource competition within the stand and affecting the optimization of stand structure.

Building on this foundation, our research team applied multi-agent reinforcement learning to integrate selective harvesting and replanting, using multiple agents for collaborative optimization. Compared to single selective harvesting or replanting, multi-agent reinforcement learning offers advantages such as improved harvesting effectiveness and more flexible replanting locations, providing high adaptability and variability (Xuan et al., 2024, 2023). However, for such complex optimization problems, the trial-and-error cost in reinforcement learning increases, leading to unstable training and poor generalization capability. In contrast, multi-agent deep reinforcement learning not only retains the collaborative optimization advantages of multi-agent reinforcement learning but also exhibits superior computational efficiency. It achieves higher solution stability and efficiency when handling complex problems, along with enhanced generalization capability (Waschneck et al., 2018; Ning et al., 2023; Gronauer and Diepold, 2022; Shen et al., 2022).

Despite significant progress in current stand structure optimization research (Olsthoorn et al., 1999; Chen et al., 2023; Zhang et al., 2024), existing studies primarily focus on optimizing the present stand condition while overlooking the dynamic changes in stand structure. Research on optimizing dynamic stand structures remains relatively scarce (Na, 2019). As climate change, ecological shifts, and increasing complexities in forest management demand more adaptive strategies, dynamic stand structure optimization—optimization from a long-term perspective—will become increasingly important. Therefore, integrating scientific prediction with effective optimization methods to enhance the sustainability and adaptability of future stand management has emerged as a key challenge in contemporary forestry research.

Structure prediction is another important field in ecology and forest management. By analyzing existing stand data, it forecasts future stand growth trends and structural evolution.

Extensive research has been conducted on predicting stand variables such as DBH (Gyawali et al., 2015; Bohora and Cao, 2014), tree height (Lee et al., 2024; Siipilehto et al., 2023), crown width (Raptis et al., 2018; Sánchez-González et al., 2007), and crown length (Mengying et al., 2021; Sattler and LeMay, 2011). General growth models (Jiazheng et al., 2021) offer biologically interpretable insights, mixed-effects models (Chang and Fan, 2024) capture both population trends and individual variation, and machine learning methods such as random forests (Xiaonan et al., 2024) excel in nonlinear modeling and predictive accuracy. These approaches have all achieved promising results. Particularly in forest management and resource planning, these models provide critical scientific support for stand management. However, existing studies on structure prediction mainly focus on the interactions and trend predictions of multiple tree attributes (Ling-bo and Zhao-gang, 2011; Xi et al., 2015), with relatively little attention given to their overall impact on stand structural evolution (Jiping et al., 2020). Integrating prediction with optimization not only enhances our understanding of the dynamic changes in forest ecosystems but also provides scientific guidance for dynamic stand structure optimization, ultimately improving the long-term effectiveness of forest management.

In summary, although existing studies have made significant progress in stand structure optimization, they primarily focus on static improvements and fail to adequately address the dynamic nature of stand development over time. Moreover, heuristic algorithms and traditional reinforcement learning methods suffer from limitations in computational efficiency and generalization ability, restricting their applicability in long-term, complex ecosystem management. To bridge these gaps, this study proposes an innovative approach that integrates multi-agent deep reinforcement learning with stand structure prediction, focusing on the secondary forests of *Pinus yunnanensis*. By incorporating dynamic prediction into selective harvesting and replanting measures, we aim to achieve dynamic optimization of stand structure, not only enhancing the current structural condition but also improving long-term stability and sustainability.

## 2 Materials and methods

### 2.1 Study areas

**Figure 1:**
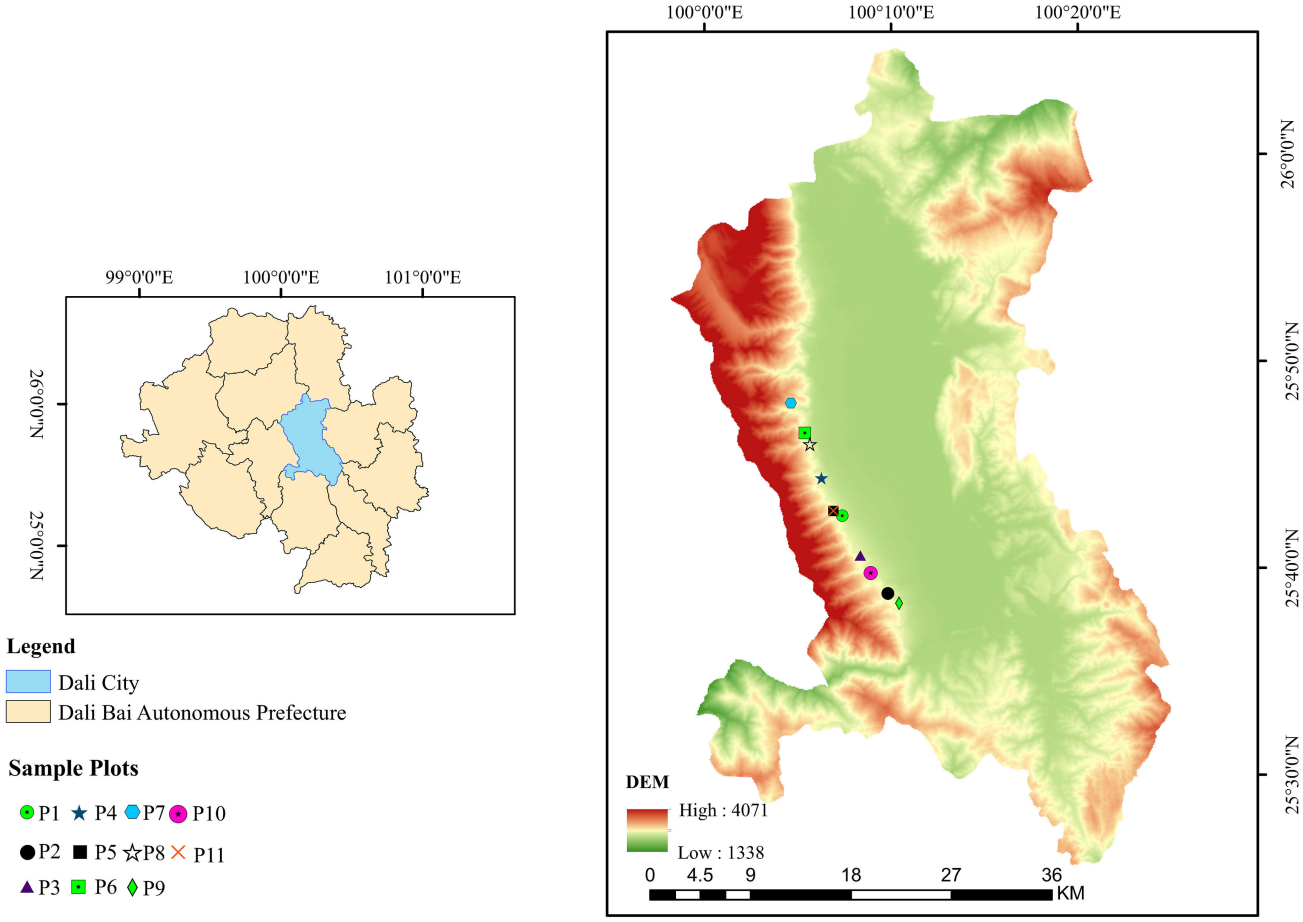
Location of the study area.

The study area is located in the Cangshan region of the Dali Bai Autonomous Prefecture, Yunnan Province. *Pinus yunnanensis* is a typical pioneer and dominant species in this region, playing a key role in water conservation, soil and water preservation, and the maintenance of biodiversity. However, due to historical overexploitation, secondary *Pinus yunnanensis* forests in this area generally exhibit simple stand structures and poor stability, making them a focus and challenge for sustainable forest management. Therefore, conducting structural optimization studies on this typical forest type is of significant theoretical and practical importance for achieving precise improvement of regional forest ecosystems. The study area is located in Cangshan, Dali, Yunnan Province, southwestern China, spanning 25°34^′^ ∼ 26°00^′^ *N*, 99°55^′^ ∼ 100°12^′^ *E*, with a total area of approximately 293 km² and an elevation range of 1966–4122 m. The region has a plateau monsoon subtropical climate characterized by mild and stable weather, ample sunlight, small annual temperature variations, and large diurnal temperature fluctuations, with an annual average temperature of 16.1°C. The prevailing wind direction is the southwest monsoon (Shuai et al., 2024). Annual precipitation is abundant, reaching 861.1 mm, with distinct dry and wet seasons. Rainfall is concentrated from May to October, accounting for 83% of the total annual precipitation. The predominant soil type in the area is Hyperdystric Clayic Ferralsol (Ferric). *Pinus yunnanensis* is the primary tree species, and the associated tree species in the canopy layer include *Pinus armandii* Franch., *Betula alnoides* Buch.-Ham. ex D. Don, *Quercus acutissima* Carruth., and *Quercus variabilis* Blume. The understory shrub layer includes species such as *Vaccinium bracteatum* Thunb., *Rhododendron microphyton* Franch., *Gaultheria griffithiana* Wight, *Eurya nitida* Korthals, and *Ternstroemia gymnanthera* (Wight & Arn.) Bedd (**Figure 1**).

### 2.2 Study site and data collection

When establishing standard sample plots, circular plots offer advantages over traditional ones, such as easier setup and positioning in complex terrains and a smaller edge effect for the same area (Packalen et al., 2023). Generally, common square plots are typically set at 20m×20m as the initial size, which translates to a circular plot with a radius of approximately 11.29m. To study the structural characteristics and optimization methods of secondary *Pinus yunnanensis* forests at different scales, this research established circular plots of varying radii based on topographic conditions and plot accessibility, in accordance with predefined rules for standard plot radius division.

Based on the terrain conditions and stand characteristics, 11 fixed circular standard plots with radii ranging from 12 to 35 meters were established at elevations between 2100 and 2400 meters on Cangshan Mountain (Packalen et al., 2023). The geographical coordinates, elevation, slope, aspect, and plot radius of each plot were measured and recorded. For each circular standard plot, all living trees with a diameter at breast height of at least 5cm were individually measured. The species, relative coordinates, DBH, tree height, crown width, and other basic tree factors were recorded for each tree. The relative coordinates of each tree at the base were accurately measured using a total station (GTS-2002). Additionally, plots with better site conditions, P1-P5, were selected as the experimental plots for simulation optimization, with the basic plot information provided in **Table 1**.

**Table 1:** Basic information of the sample plots.

Sample plots	Altitude	Slope (°)	Slope dir.	Mean DBH (cm)	Mean height (m)	Sample plot radius (m)	Survey time	Stand density (trees· ha^−1^)
P1	2254	13.45	E	17.10	11.97	35	2022	1603
P2	2273	16.16	S	13.79	9.39	32	2022	2182
P3	2205	17.70	NE	14.50	9.30	20	2022	2109
P4	2138	5.10	NE	14.26	10.94	19	2021	2618
P5	2253	15.25	SE	16.03	9.57	30	2023	2631
P6	2226	30.75	E	12.77	9.48	14	2021	3839
P7	2184	36.80	E	15.62	8.99	12	2021	1415
P8	2393	26.30	NE	21.33	13.58	12	2021	1627
P9	2136	13.60	NE	15.51	9.41	15	2021	1627
P10	2194	11.75	S	16.17	9.88	19	2022	2504
P11	2284	30.35	SE	15.69	8.58	18	2022	2504

E, east; S, south; NE, north-east; SE, south-east.

### 2.3 Determination of spatial structure units and edge correction

This study employed the Voronoi diagram method to determine spatial relationships among trees (Liu et al., 2023). Centered on a reference tree, the Voronoi diagram method accurately captures tree adjacency relationships while effectively reflecting their horizontal distribution pattern. During data processing, Voronoi diagrams were generated using R 4.2.0, with each polygon representing a spatial structural unit formed by a tree and its neighboring trees. To minimize errors in calculating spatial structure indexes caused by edge trees being fragmented at the plot boundary, this study adopted the buffer zone method. The plot boundary was contracted inward by 2 m to create a buffer zone (Von Gadow et al., 2003). When computing spatial structure indexes, trees within the buffer zone were only considered as neighboring trees for constructing spatial structural units and were not used as reference trees.

### 2.4 Stand structure indexes

Quantifying stand structure is a fundamental aspect of stand structure optimization. In this study, spatial structure was set as the primary objective, while non-spatial structure served as a constraint. The selected non-spatial structure indexes included tree diameter classes, number of species, canopy density, harvesting intensity, and planting density. The selected spatial structure indexes included the uniform angle index, complete mingling, crown competition index, stratification index, and neighbourhood comparison. Among these, the uniform angle index describes the horizontal distribution pattern of trees, complete mingling represents the degree of tree species segregation, the crown competition index quantifies competition pressure among trees, the stratification index characterizes the vertical distribution pattern, and neighborhood comparison measures the degree of size differentiation among trees.

#### 2.4.1 Non-spatial structure indexes

##### 2.4.1.1 Tree diameter classes

Trees are classified into different categories based on their DBH, with a greater number of diameter classes indicating better stand growth. In the optimization process, it is required that the diversity of tree diameter classes remains consistent before and after optimization. In this study, tree diameter classification starts from a DBH of 6 cm, with a 2 cm interval for each diameter class (Equation 1).


(1) $$D=D_{0}$$

Where *D*_0_ represents the number of diameter classes of trees within the stand before harvesting, and *D* represents the number of diameter classes of trees within the stand after harvesting.

##### 2.4.1.2 Number of species

During the optimization process, tree species diversity must be preserved, and no species should be artificially eliminated from the stand. It is required that the tree species diversity remains consistent before and after optimization to ensure that no species disappear (Equation 2).


(2) $$T=T_{0}$$

Where *T*_0_ denotes the initial number of tree species, while *T* indicates the number of tree species after harvesting.

##### 2.4.1.3 Canopy density

A healthy forest requires the canopy to form a continuous cover. Generally, a canopy density of no less than 0.7 is considered indicative of continuous forest cover (Equation 3).


(3) Cd≥0.7

##### 2.4.1.4 harvesting intensity

harvesting intensity determines whether the stand’s growth condition remains favorable after optimization. According to harvesting requirements, the amount of selective harvesting should be less than the growth increment. Research indicates that the harvesting intensity of *Pinus yunnanensis* secondary forests should be controlled within 35% (Su et al., 2010; Han et al., 2011) (Equation 4).


(4) $$N\geq N_{0}(1-35\%)$$

Where *N*_0_ represents the total number of trees before harvesting, while *N* represents the total number of trees after harvesting.

##### 2.4.1.5 Planting density

The planting density is a key factor influencing the effectiveness of replanting. Previous studies have shown that the optimal planting density for Pinus yunnanensis ranges from 1667 to 3333 trees per hectare (Zhang et al., 2023). After replanting optimization, the stand density should fall within the range of [1,667, 3,333] trees per hectare (Equation 5).


(5) 1667≤PD≤3333

#### 2.4.2 Spatial structure indexes

##### 2.4.2.1 Neighborhood comparison (*U*)

Neighborhood comparison (Aguirre et al., 2003) is used to describe the degree of size differentiation and competition among trees. It refers to the proportion of neighboring trees with a DBH larger than that of the reference tree among neighboring trees. The expression is given as (Equation 6):


(6) $$U_{i}=\frac{1}{n}\sum_{j=1}^{n}k_{ij}$$

*U_i_*represents the neighborhood comparison for reference tree *i*, If the diameter at breast height of neighboring tree *j* is greater than that of reference tree *i*, then *k_ij_* = 1, otherwise, *k_ij_* = 0. A smaller *U_i_* indicates a greater dominance of the reference tree. The value of *U_i_* can fall into five intervals: 0, (0, 0.25], (0.25, 0.5], (0.5, 0.75], and (0.75, 1], corresponding to the reference tree being in dominant, sub-dominant, intermediate, disadvantaged, and absolutely disadvantaged status within the stand, respectively.

##### 2.4.2.2 Crown competition index

The crown competition index (Jianming, 2017) is a method used to describe the degree of competition among trees by calculating the crown overlap area based on tree characteristics such as crown width and crown length, thereby reflecting the competitive pressure during tree growth. The expression is given as (Equations 7–10):


(7) $$CI_{i}=\frac{1}{Z_{i}}\times \sum_{j=1}^{n}AO_{ij}\times \frac{L_{j}}{L_{i}}$$

*CI_i_* represents the crown competition index for reference tree *i*, and *Z_i_* represents the crown projection area of reference tree *i*. *L_j_*= *H_j_* × *CW_j_* × *CL_j_* (height of competing tree *j* × crown width of competing tree *j* × crown length of competing tree *j*), *L_i_*= *H_i_*× *CW_i_*× *CL_i_* (height of reference tree *i* × crown width of reference tree *i* × crown length of reference tree *i*). *AO_ij_* represents the crown overlap area between reference tree *i* and competitor tree *j*. If there is no overlap, *AO_ij_* = 1. When there is overlap,


(8) $$S_{0}=\frac{CW_{i}^{2}}{2}\sum_{j=1}^{n}arccos(\frac{q_{j}^{2}}{2CW_{i}^{2}}-1)-\frac{1}{4}q_{j}\sqrt{4CW_{i}^{2}-q_{i}^{2}}$$

(9) $$S_{1}=\frac{1}{2}\sum_{j=1}^{n}\{[CW_{j}^{2}arccos(1-\frac{4CW_{i}^{2}-q_{i}^{2}}{2CW_{j}^{2}})-\frac{\sqrt{4CW_{i}^{2}-q_{j}^{2}}}{2}]\times \sqrt{4CW_{i}^{2}-(4CW_{i}^{2}-q_{j}^{2})}\}$$

(10) $$AO_{ij}=S_{0}+S_{1}$$

*S*_0_ represents the total shaded area of reference tree *i* by *n* competitor trees, and *S*_1_ represents the total shaded area of *n* competitor trees by reference tree *i*. $q_{j}=\frac{L_{ij}^{2}-(CW_{j}^{2}-CW_{i}^{2})}{L_{ij}},$ *L_ij_* represents the distance between competitor tree *j* and reference tree *i*, *CW_i_* represents the crown width of reference tree *i*, *CW_j_* represents the crown width of competitor tree *j*, and *n* represents the number of competitor trees.

##### 2.4.2.3 Stratification index

The stratification index (Zhao et al., 2024; Zhou et al., 2022) reflects the vertical distribution pattern of trees and the diversity of stand structure. It is an extension of the storey index, incorporating the influence of terrain on forest stratification. The expression is given as (Equations 11–13):


(11) $$S_{i}=\frac{z_{i}}{3}\times \frac{1}{n}\sum_{j=1}^{n}(1-\frac{\left|FL_{i}-FL_{j}\right|}{max(|FL_{i}-FL_{j}|,\;1)})$$

(12) $$FL_{i}=\{\begin{array}{l} -1,H_{i}\leq \frac{1}{3}H_{d}\\ 0,\frac{1}{3}H_{d}\leq H_{i}\leq \frac{2}{3}H_{d}\\ 1,H_{i}\geq \frac{2}{3}H_{d} \end{array}$$

(13) $$H_{d}=\frac{1}{\left\lfloor 100A\right\rfloor }\sum_{i=1}^{\left\lfloor 100A\right\rfloor }(H_{sort(i)}+E_{sort(i)})$$

*S_i_*represents the stratification index for reference tree *i*, *z_i_* denotes the number of layers within the spatial structure unit to which the reference tree *i* belongs. *FL_i_* indicates the classification of reference tree *i* in the vertical stratification. *H_i_* represents the height of reference tree *i*, while *H_d_* denotes the dominant height. *A* stands for the per hectare plot area, and *H_sort_*_(_ *_i_*_)_ is the height of the *i*-th tree among the tallest ⌊100*A*⌋ trees per hectare, and *E_sort_*_(_ *_i_*_)_ indicates the relative elevation of the *i*-th tree among these ⌊100*A*⌋ trees. The closer the stratification index is to 1, the more complex the vertical stratification of the stand.

##### 2.4.2.4 Complete mingling

Complete mingling (Sheng et al., 2023) introduces the Simpson index into the traditional mingling index to enhance the differentiation of tree species diversity. It is used to describe the degree of tree species segregation while also accounting for species diversity. The expression is given as (Equation 14):


(14) $$Mc_{i}=\frac{M_{i}}{2}[1-\frac{1}{{\left(n+1\right)}^{2}}\sum_{j=1}^{s_{i}}n_{j}^{2}+\frac{n_{i}}{n}]$$

*Mc_i_* represents the complete mingling of reference tree *i*. *n_j_* is the number of different species among the neighboring trees, *n_j_* is the number of trees of the *j*-th species among the neighboring trees, and *s_i_*is the number of species within the spatial structure unit to which reference tree *j* belongs. *M_i_* represents the mingling degree of reference tree *i* $M_{i}=\frac{1}{n}\sum_{j=1}^{n}v_{ij}$ , When the reference tree *i* and neighboring tree *j* are of the same species, *v_ij_* = 0; otherwise, *v_ij_* = 1. The value of *Mc_i_* can fall into five intervals: 0, (0, 0.25], (0.25, 0.5], (0.5, 0.75], and (0.75, 1], corresponding to zero mixing, low mixing, moderate mixing, high mixing, and complete mixing, respectively.

##### 2.4.2.5 Uniform angle index (*W*)

The uniform angle index (Zhang et al., 2018) is used to describe the spatial distribution pattern of trees. It is defined as the proportion of α angles (the smaller angles between neighboring trees) that are less than the standard angle $\alpha_{0}(\alpha_{0}=\frac{360^{\circ }}{n+1})$ out of a total of angles formed. Its expression is (Equation 15):


(15) $$W_{i}=\frac{1}{n}\sum_{j=1}^{n}z_{ij}$$

*W_i_* represents the uniform angle index for reference tree *i*. When the *j*-th *α* angle is smaller than the standard angle *α*_0_, *z_ij_* = 1; otherwise, *z_ij_* = 0. The value of *W_i_* can fall into five intervals: 0, (0, 0.25], (0.25, 0.5], (0.5, 0.75], and (0.75, 1], corresponding to absolutely uniform, uniform, random, non-uniform, and conmplete non-uniform distributions, respectively. The ideal range for the mean uniform angle index in a stand is between [0.475, 0.517].

### 2.5 Selective harvesting Strategy

Random selection (Tang et al., 2004), tree homogeneity index (Yitong, 2019), and spatial competition (Zhang et al., 2019) are common methods for determining felling decisions. In the random selection method, trees are randomly selected as candidates for felling from the initial stand. The tree homogeneity index-based method calculates a comprehensive index Li for each tree using spatial structure parameters and ranks the trees in ascending order to determine the felling candidates. The spatial competition-based method evaluates trees based on horizontal spatial patterns and competition pressure, selecting trees with a greater difference between the uniform angle index and 0.496, a higher neighborhood comparison value, and a larger crown competition index as felling candidates. In our previous research, we conducted an experimental comparison of these three methods and found that random selection was best suited for integration with the deep reinforcement learning algorithm Zhao et al. (2024). Therefore, in this study, random selection is chosen as the preferred method for the felling optimization process.

### 2.6 Replanting strategy

#### 2.6.1 Planting location

The maximum Delaunay triangulation area method is a planting location determination approach based on Delaunay triangulation. In a Delaunay triangulation network, edge lengths represent the distances between neighboring trees, while nodes correspond to individual trees. Planting locations are determined by identifying the incenter of the largest triangle formed by the nodes, thereby reflecting both the presence of canopy gaps and the overall stand distribution pattern. To account for the influence of surrounding neighboring trees on the growth of replanted trees, the replanting foreground index (RFI) (Xuan et al., 2024) is incorporated to identify planting locations that have a greater impact on stand structure optimization. This method comprehensively considers various factors affecting the growth of replanted trees, thereby improving the accuracy and effectiveness of planting location determination and enhancing the efficiency of stand structure adjustment and optimization.

The calculation formula for the RFI is as follows (Equation 16):


(16) $$RFI_{i}=\frac{\frac{1+DAA_{i}}{\delta_{DAA}}\cdot \frac{1+Mc_{i}}{\delta_{Mc}}\cdot \frac{1+U_{i}}{\delta_{U}}}{\frac{1+CI_{i}}{\delta_{CI}}}$$

*RFI_i_* represents the replanting foreground index of the replanted tree *i*, *DAA_i_* refers to the area of the Delaunay triangulation element in which tree *i* is located. *Mc_i_* corresponds to the complete mingling of tree *i*, while *U_i_* denotes its neighborhood comparison, and *CI_i_* indicates its crown competition index. Moreover, *δ_DAA_*, *δ_Mc_*, *δ_U_* and *δ_CI_* represent the standard deviations of their respective structural parameters.

#### 2.6.2 Planting number

Relevant studies have shown that the optimal planting density range for *Pinus yunnanensis* is 1667–3333 trees per hectare. In this study, 3333 trees per hectare was selected as the upper limit for stand density after replanting. Based on the plot area, the maximum number of trees was determined for each plot: P1, P2, P3, P4, and P5 had upper limits of 1284, 1076, 418, 377, and 941 trees, respectively.

#### 2.6.3 Spatial configuration of replanting

In mixed forests, the proportion and spatial arrangement of replanted tree species directly influence the degree of species mingling, while variations in DBH, H, and CW among different species substantially affect interspecific competition. In this study, replanting optimization simultaneously considered the spatial distribution and size structure of trees to achieve a more balanced species composition and stand structure. To promote the positive succession of secondary forests and enhance the mingling index, the main tree species widely distributed in the Cangshan region were selected. The seven chosen species—*Pinus yunnanensis*, *Pinus armandii*, *Quercus acutissima*, *Vaccinium bracteatum*, *Camellia sinensis*, *Betula alnoides*, and *Ternstroemia gymnanthera*—are either dominant or native tree species in the study area and represent the typical species composition and ecological characteristics of local secondary forests. Equal proportions were applied in the simulation to simplify the modeling framework and highlight species diversity and ecological complementarity. The replanted trees were initialized with an average DBH of 5 cm and an age of 5 years. Due to the limited availability of growth models for some species, models of ecologically similar species (Xi et al., 2015; Luo, 2021; Jiazheng et al., 2021) were adopted to estimate tree height, crown width, and crown length, and the results were subsequently validated using existing datasets. The configuration of replanted trees is shown in **Table 2**.

**Table 2:** Configuration of replanted trees.

Tree species	Variable	Model	Optimal parameters	Stand factor
*Pinus yunnanensis*	H	$H=a\times {\left(1-e^{-b\times DBH}\right)}^{c}$	*a* = 16.7289, *b* = 0.0871, *c* = 1.1212	H=5.20
	CW	$CW=a\times DBH^{b}$	a=0.5652,b=0.7023	CW=1.74
	CL	$CL=a\times DBH^{b}$	a=0.3646,b=0.7984	CL=1.32
*Pinus armandii*	H	$H=a\times {\left(1-e^{-b\times DBH}\right)}^{c}$	*a* = 17.0986, *b* = 0.0816, *c* = 0.9258	H=5.19
	CW	$CW=\frac{a}{1+b\times e^{-c\times DBH}}$	*a* = 4.2420, *b* = 2.2677, *c* = 0.0523	CW=1.54
	CL	$CL=a\times {\left(1-e^{-b\times DBH}\right)}^{c}$	*a* = 41.0291, *b* = 0.0289, *c* = 0.4971	CL=2.56
*Quercus acutissima*	H	$H=a\times e^{b+c/(DBH+1)}+d$	*a* = 1.0822, *b* = 3.2453, *c* = 17.2914, *d* = 3.5632	H=5.12
	CW	$CW=a+b\times DBH^{2}+c\times DBH^{3}$	*a* = 0.2574, *b* = 0.2442, *c* = –0.0022	CW=1.82
	CL	$CL=a\times DBH^{b}$	a=0.4833,b=0.6892	CL=1.47
*Betula alnoides*	H	$H^{-1}=a+b\times DBH$	a=0.0211,b=0.6850	H=6.33
	CW	$CW=\frac{a}{1+b\times e^{-c\times DBH}}$	*a* = 3.0360, *b* = 0.8446, *c* = 0.1345	CW=2.12
	CL	$CL=a\times DBH^{b}$	a=2.0317,b=0.2375	CL=2.98
*Vaccinium bracteatum*	H	$H=a\times e^{-b/DBH^{c}}$	*a* = 2.2004, *b* = 0.5011, *c* = 4.9997	H=2.20
	CW	$CW=\frac{a}{1+b\times e^{-c\times DBH}}$	*a* = 2.9716, *b* = 1.0269, *c* = –0.0314	CW=1.35
	CL	$CL=\frac{a}{1+b\times e^{-c\times DBH}}$	*a* = 1.0123, *b* = 0.7829, *c* = 4.0326	CL=1.01
*Camellia sinensis*	H	$H=a\times {\left(1-e^{-b\times DBH}\right)}^{c}$	*a* = 10.0002, *b* = 0.2114, *c* = 1.9997	H=4.26
	CW	$CW=\frac{a}{1+b\times e^{-c\times DBH}}$	*a* = 1.9389, *b* = 0.5366, *c* = 0.1097	CW=1.48
	CL	$CL=a\times DBH^{b}$	a=0.9009,b=0.3250	CL=1.55
*Ternstroemia gymnanthera*	H	$H=a+b\times DBH+c\times DBH^{2}$	*a* = 2.9963, *b* = 0.9818, *c* = –0.1110	H=5.13
	CW	ln(CW)=a+b×ln(DBH)	a=−0.1710,b=0.2247	CW=1.21
	CL	$CL=a\times {\left(1-e^{-b\times DBH}\right)}^{c}$	*a* = 1.9964, *b* = 0.1762, *c* = 1.0005	CL=1.17

### 2.7 Stand structure prediction

#### 2.7.1 Models

Based on the data characteristics of different tree factors and the requirements of the prediction tasks, this study selected four different machine learning models to ensure predictive accuracy and reliability. The CNN-PSO (Ma et al., 2022) model is suitable for handling continuous variables with complex nonlinear relationships; its convolutional layers can effectively capture latent spatial patterns among features, and thus it was used to predict age and crown length. The Random Forest (RF) model (Xiaonan et al., 2024), with its strong anti-overfitting capability and ability to handle high-dimensional data, can robustly manage complex interactions among tree growth factors, making it suitable for predicting tree height and crown width. The Multilayer Perceptron (MLP) (Kim et al., 2025), due to its powerful function approximation capability, was employed to model the growth of diameter at breast height (DBH), a complex nonlinear regression problem. The Gradient Boosting Decision Tree (GBDT) (Nhat-Duc and Van-Duc, 2023), which excels in classification tasks—particularly with imbalanced data and combining multiple weak classifiers—was used to predict the probability of tree mortality, a binary classification problem. This targeted model selection strategy aims to maximize the accuracy and robustness of each prediction task.

##### 2.7.1.1 CNN-PSO

CNN consists of an input layer, output layer, convolutional layers, pooling layers, and fully connected layers. Its core concept is to automatically learn local features through convolutional layers, eliminating the need for manual feature extraction required by traditional methods. Additionally, pooling layers perform downsampling on the features, reducing computational complexity while enhancing the model’s robustness.

PSO is a global optimization algorithm that simulates swarm behavior, where each particle represents a potential solution. The particles continuously move through the solution space, searching for the optimal solution. PSO has strong global search capabilities and can efficiently identify high-quality solutions.

The CNN-PSO was adopted in this study, where the global search ability of PSO was utilized to optimize critical hyperparameters, such as the number of output channels in the convolutional layers, learning rate, and batch size. Traditional manual tuning is often constrained by experience and prone to getting stuck in local optima. By applying PSO to CNN hyperparameter optimization, the optimal parameter combination can be automatically identified, enhancing model performance while significantly reducing computational resources and time.

**Figure 2:**
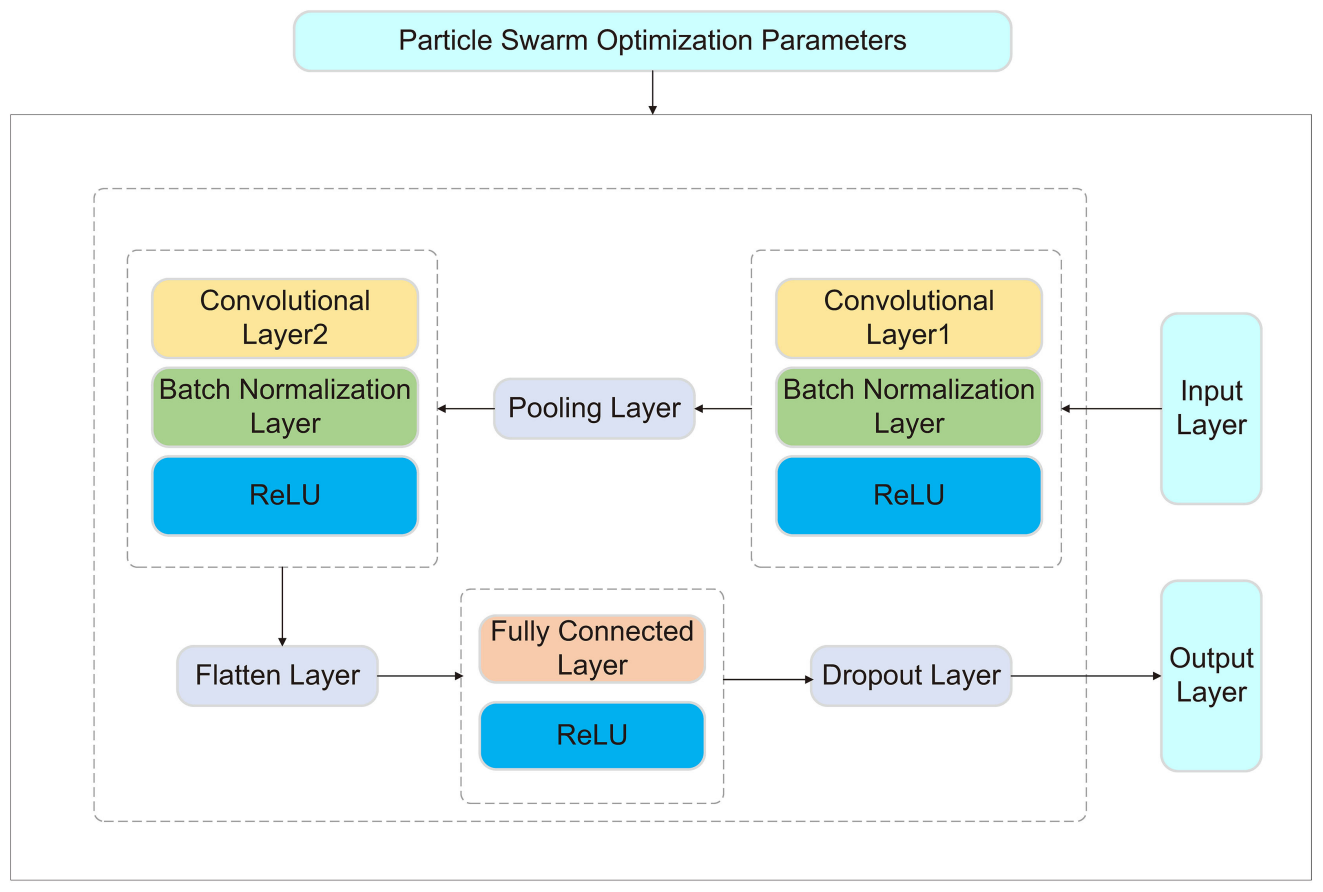
The architecture of the CNN-PSO model.

In the model design, two convolutional layers were used to extract features from the input data, each followed by a batch normalization layer to accelerate training and improve stability. Then, pooling layers were applied to reduce data dimensionality, alleviating computational burden and enhancing the model’s adaptability to local features. After convolution and pooling, the data was flattened and processed through fully connected layers before producing the final prediction output. To prevent overfitting, a Dropout layer was incorporated to randomly drop some neurons, encouraging the model to learn diverse features and thereby improving its predictive capability on new data. As shown in **Figure 2**.

##### 2.7.1.2 Random Forest

RF is an ensemble learning algorithm based on the Bagging method and decision trees, using decision trees as its fundamental units. It constructs multiple decision trees and combines their predictions to enhance overall performance. During training, each tree is built using a randomly sampled subset of the original data, and feature selection at each node split is performed randomly. This approach reduces overfitting and improves the model’s generalization ability. By integrating the outputs of multiple decision trees, random forest effectively captures complex feature interactions, exhibits strong noise resistance, and achieves high prediction accuracy.

**Figure 3:**
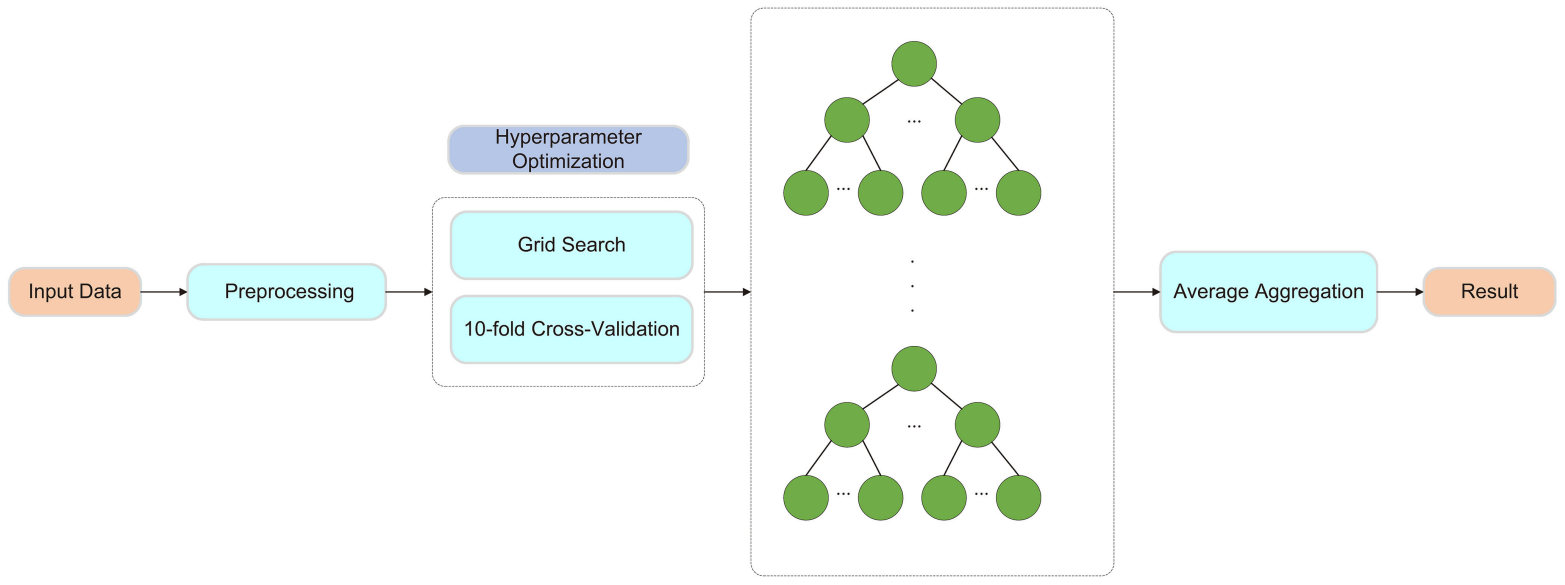
Framework of the Random Forest Model.

To further enhance the predictive performance of the model, this study employed ten-fold cross-validation and grid search for hyperparameter optimization. Ten-fold cross-validation involves partitioning the dataset into ten subsets, using nine for training and the remaining one for validation in each iteration. This method effectively mitigates biases caused by data partitioning and improves the model’s stability and generalization ability. Grid search systematically explores different hyperparameter combinations to optimize the performance of the random forest model. The tested hyperparameters include the number of trees (50, 100, 200), tree depth (10, 20, 30), the minimum number of samples required for node splitting (2, 5, 10), and the minimum number of samples required for leaf nodes (1, 2, 4). As shown in **Figure 3**.

##### 2.7.1.3 Multilayer perceptron

MLP is a deep feedforward neural network composed of an input layer, multiple hidden layers, and an output layer. Each neuron in a layer is fully connected to the neurons in the previous layer. Input data undergoes weighted summation and activation function processing at each layer, ultimately producing the final prediction output. MLP possesses strong function approximation capabilities, enabling it to capture complex patterns and features in data through multiple hidden layers, thereby achieving high predictive accuracy.

**Figure 4:**
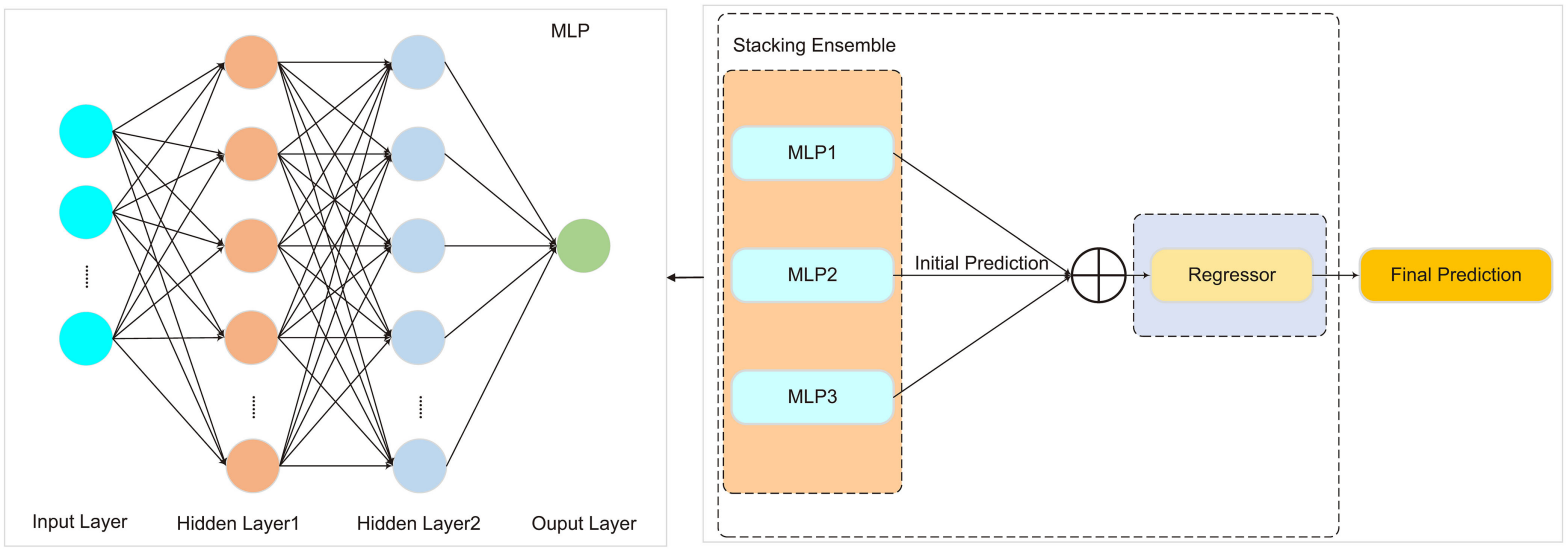
The architecture of the Stacked-MLP model.

In the model design, this study utilized MLP as the base model and constructed a Stacked-MLP model by stacking three MLPs. The prediction outputs of each MLP were used as inputs for a regressor, which generated the final prediction. To balance computational cost and model performance, the hyperparameters of the regressor were set as follows: learning rate (0.0001–0.1), number of iterations (100–500), and batch size (16, 32, 64, 128). As shown in **Figure 4**.

##### 2.7.1.4 Gradient boosting decision tree

GBDT is an ensemble learning-based classification algorithm that incrementally trains multiple weak classifiers, combining their weighted predictions to obtain the final classification result. Each new model is optimized to correct the errors made by the previous model, focusing on misclassified samples and gradually adjusting the model parameters to improve classification performance. GBDT efficiently handles complex nonlinear relationships and can automatically identify interactions between features, reducing the need for extensive manual feature engineering. It offers high predictive accuracy and strong flexibility, making it a powerful tool for classification tasks.

**Figure 5:**
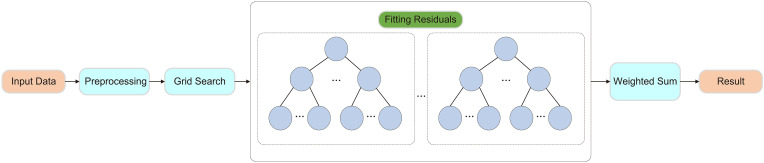
Framework of the Gradient Boosting Decision Tree Model.

To enhance model performance, improve stability, and strengthen generalization ability, this study employed grid search to explore the hyperparameter space and identify the optimal combination of parameters. The selected hyperparameters include learning rate (0.01, 0.1, 0.2), number of trees (50, 100, 200), and tree depth (10, 20, 30). As shown in **Figure 5**.

#### 2.7.2 Prediction

Stand structure prediction involves forecasting parameters such as diameter at breast height, tree height, age, crown width, crown length, and mortality rate to understand the dynamic changes in stand structure. This enables dynamic optimization of stand structure and provides decision-making support for forest management, resource conservation, and ecological restoration.

We selected various indicators to construct the model, including age (*AGE*), diameter at breast height (*DBH*,1*/DBH,DBH* ^2^), tree height (*H*), crown width (*CW*), crown length (*CL*), number of trees per hectare (*NT*), stand density index (*SDI*), slope (*SLO*), aspect (*ASP*), height-to-diameter ratio (*HDR*), the sum of basal area larger than the target tree (*BAL*), and Hegyi competition index (*HCI*). To eliminate multicollinearity when building different predictive models, independent variables with a variance inflation factor (VIF) greater than 10 were excluded.

The diameter growth model was developed using the Stacked-MLP algorithm, with *AGE*, *ASP*, *NT*, *SLO*, *SDI*, *BAL*, and *DBH* ^2^ selected as feature variables. The model’s target variable was set as the natural logarithm of diameter growth squared plus one, *ln*(*DGI* + 1). Since tree diameter growth occurs over long periods, shorter prediction intervals may fail to capture significant growth changes. Therefore, a five-year prediction cycle was used in the model.

The RF algorithm was used for tree height and crown width prediction models. The selected feature variables for tree height prediction were *AGE*, *ASP*, *DBH*, and *SLOPE*, with *H* as the target variable. For crown width prediction, the feature variables included *AGE*, 1*/DBH*, *CL*, *ASP*, *SDI*, *HDR*, and *NT*, with *CW* as the target variable.

The CNN-PSO was used for age and crown length prediction models. The feature variables for age prediction were 1*/DBH*, *H*, *ASP*, *SLO*, *NT*, and *SDI*, with *AGE* as the target variable. For crown length prediction, the selected feature variables were *AGE*, 1*/DBH*, *ASP*, *SDI*, *HDR*, and *NT*, with *CL* as the target variable.

Mortality prediction is a binary classification problem, and the GBDT model was chosen for this task. To improve prediction accuracy, the classification threshold was initially set at 0.5; however, this value is only applicable when the number of surviving and dead trees in the stand is approximately equal. In reality, mortality is a low-probability event, and cases where mortality and survival are equal are rare. Therefore, this study incorporated the HCI as an additional criterion. Trees with a mortality probability greater than 0.5 and experiencing high competition pressure (*HCI >* 0.75) were classified as dead. The selected feature variables included 1/DBH, H, BAL, and HCI, with tree mortality status (*STATE*) used as the target label.

Since the stand structure prediction model simultaneously predicts multiple stand factors, redundancy in feature variable selection is inevitable, which may lead to internal inconsistencies within the model. To address this, predictions were conducted sequentially in the following order: age, diameter at breast height, tree height, mortality, crown width, and crown length. This ensures that the feature variables used in all prediction models are updated accordingly during the prediction process.

The VIF values of the feature variables and the prediction accuracy of each model are presented in **Tables 3**–**5**.

**Table 3:** VIF values of feature variables in different prediction models.

Age prediction		DBH prediction		H prediction		CW prediction		CL prediction		Mortality prediction	
feature	VIF	feature	VIF	feature	VIF	feature	VIF	feature	VIF	feature	VIF
1/DBH	1.5352	DBH	1.4852	DBH	4.6597	AGE	5.8744	AGE	3.5899	$DBH^{2}$	4.1586
H	1.6049	AGE	2.0224	AGE	4.6514	1/DBH	4.4287	1/DBH	4.4283	H	5.8075
ASP	2.1709	ASP	2.0374	ASP	1.3759	CL	2.0131	ASP	2.2043	BAL	2.0631
SLO	1.1583	SLO	1.2576	SLO	1.3651	ASP	2.2399	SDI	2.3709	HCI	1.1315
NT	1.5428	NT	1.2727			HDR	1.6552	HDR	1.5049		
SDI	2.5316	BAL	1.5647			NT	1.0445	NT	1.0434		
		SDI	2.5414			SDI	2.3858				

**Table 4:** Evaluation metrics of selected prediction models.

Target variable	Model	*R* ^2^	*RMSE*	*MAE*
*AGE*	CNN-PSO	0.4246	5.3775	4.1626
*DBH*	Stacked-MLP	0.8461	0.3055	0.2041
*H*	RF	0.8280	1.4143	1.0992
*CW*	RF	0.6315	0.3554	0.2704
*CL*	CNN-PSO	0.6403	1.02485	0.7951

**Table 5:** Evaluation metrics for the mortality prediction model.

Target variable	Model	*Acc*	*F*1*Score*	*Recall*	*ROCAUC*
*Mortality*	GBDT	0.9002	0.7850	0.7287	0.9486

### 2.8 Optimization model of stand structure

#### 2.8.1 Constrain

After optimization adjustments, the values of each sub-objective must not fall below their pre-optimization levels to ensure that the diversity of stand spatial structure does not decline. This means that the horizontal distribution pattern of the stand should become closer to a random distribution, the degree of species mingling should increase, the richness of vertical structure should be enhanced, and both size differentiation and competition pressure should be correspondingly reduced. During the optimization process, the number of tree diameter classes and species should not decrease, harvesting intensity should be controlled within 35%, and canopy density should not fall below 0.7. The number of replanted trees should remain within the stand density range, and after replanting, both the degree of mingling and the horizontal distribution pattern should be improved compared to prereplanting conditions (Equation 17).


(17) $$s.t.\{\begin{array}{l} \bar{Mc_{1}}\geq \bar{Mc_{0}}\\ \bar{S_{1}}\geq \bar{S_{0}}\\ \bar{U_{1}}\leq \bar{U_{0}}\\ \bar{CI_{1}}\leq \bar{CI_{0}}\\ \left|{\bar{W}}_{1}-0.496|\leq |{\bar{W}}_{0}-0.496\right|\\ D_{1}=D_{0}\\ T_{1}=T_{0}\\ Cd\geq 0.7\\ N_{1}\geq N_{0}(1-35\%)\\ \left|{\bar{W}}_{2}-0.496|\leq |{\bar{W}}_{1}-0.496\right|\\ \bar{Mc_{2}}\geq \bar{Mc_{1}}\\ 1667\leq PD\leq 3333 \end{array}$$

$\bar{Mc_{1}}$ , $\bar{S_{1}}$ , $\bar{U_{1}}$ , $\bar{CI_{1}}$ , $\bar{W_{1}}$ , $D_{1}$ , $T_{1}$ , Cd , $N_{1}$ respectively represent the values of complete mingling, stratification index, neighborhood comparison, crown competition index, uniform angle index, tree diameter classes, number of species, canopy density, and harvesting intensity after selective harvesting. $\;\bar{Mc_{0}}$ , $\bar{S_{0}}$ , $\bar{U_{0}}$ , $\bar{CI_{0}}$ , $\bar{W_{0}}$ , $D_{0}$ , $T_{0}$ , Cd , $N_{0}$ represent the values of the initial stand for the following indicators. $\bar{Mc_{2}}$ , $\bar{W_{2}}$ represent the values of after complete mingling and uniform angle index after replanting. PD represents the stand density after selective redharvesting and replanting optimization.

#### 2.8.2 Model construction

Stand structure optimization is a multi-objective problem that typically involves multiple interrelated goals. Since these objectives are often mutually constraining, it is difficult to achieve their individual optima simultaneously. Therefore, it is necessary to adopt an integrated approach and balance multiple objectives from the perspective of the overall stand. In the optimization process, the complete mingling and stratification index should be increased to enhance stand diversity and stability; the crown competition index and neighborhood comparison should be reduced to alleviate competition among individuals and prevent excessive dominance by a few large trees; and the uniform angle index should be maintained within the range close to random distribution to ensure a reasonable horizontal pattern. Accordingly, in this study, five spatial structure parameters—uniform angle index, neighborhood comparison, complete mingling, stratification index, and crown competition index—were incorporated into a multi-objective optimization model using a multiplicative–divisive approach to construct the objective function (Equation 18):


(18) $$maxL=\frac{1}{n}\sum_{i=1}^{N}\frac{\frac{1+Mc_{i}}{\delta_{Mc}}\cdot \frac{1+S_{i}}{\delta_{S}}}{\frac{1+U_{i}}{\delta_{U}}\cdot \frac{1+CI_{i}}{\delta_{CI}}\cdot \frac{1+|W_{i}-0.496|}{\delta_{\left|W_{i}-0.496\right|}}}$$

*W_i_*, *Mc_i_*, *S_i_*, *U_i_* and *CI_i_* represent the uniform angle index, complete mingling, stratification index, neighborhood comparison, and crown competition index of the reference tree, respectively. *δ_W_*, *δ_M_c*, *δ_S_*, *δ_W_*and *δ_CI_* are the standard deviations of these structural indexes. *N* represents the total number of trees in the forest stand.

### 2.9 Solution algorithm

#### 2.9.1 Multi-agent deep reinforcement learning

This study selected multi-agent deep Q-network (MADQN) as the solution algorithm. The MADQN model incorporates deep Q-network (DQN) and multi-agent Q-learning (MAQL). Each agent utilizes a deep neural network to approximate the Q-value function, enabling decision-making in large-scale and complex state spaces. By leveraging experience replay and target networks, the model achieves faster convergence, avoids overfitting, and ensures policy stability and generalization capability.

In the application of MADQN for stand structure optimization, selective harvesting and replanting serve as two key regulatory measures. Two agents, Agent1 and Agent2, were designed to achieve optimization. Each agent has its own tasks and objectives, interacting with the environment while also collaborating with each other. Through continuous learning, they work together to optimize the objective function of stand structure.Agent1 selects trees for selective harvesting based on the random selection method and adjusts its strategy according to the impact of tree removal on the objective function value. After harvesting, if the selected trees result in an increase in the objective function value, Agent1 receives a reward; otherwise, it is penalized. Through this process, Agent1 gradually learns how to select trees for harvesting, aiming to minimize unnecessary losses while maximizing the improvement of the stand’s objective function during the harvesting process.

Agent2 determines the number of replanted trees and their distribution based on the stand condition after Agent1 has completed selective harvesting and received its reward. Its goal is to compensate for gaps created by harvesting and introduce appropriate tree species to optimize stand structure, enhancing growth potential and ecological benefits. After harvesting, Agent2 utilizes the RFI to identify suitable planting locations. It then applies a curve trend-based approach (Xuan et al., 2024) to optimize the number of replanted trees. Specifically, it selects three evenly spaced replanting densities and evaluates their corresponding objective function values to establish a trend curve. Based on this trend, Agent2 adjusts the number of replanted trees by selecting new values with the same spacing before and after the current replanting density. This approach leverages curve monotonicity and extremum detection to assign rewards or penalties, refining the sampling strategy to gradually determine the optimal number of replanted trees. This ensures that under the given harvesting conditions, the replanting effect is maximized for optimal stand structure recovery.

Agent1 and Agent2 are interdependent and interact throughout the optimization process. Agent1’s selective harvesting decisions directly influence Agent2’s replanting strategy, while Agent2’s replanting results, in turn, affect the post-harvesting stand condition and consequently impact the objective function value. Through a reward and penalty mechanism, both agents continuously adjust their strategies, enabling a coordinated optimization process that dynamically balances selective harvesting and replanting to achieve the best possible stand structure.

**Figure 6:**
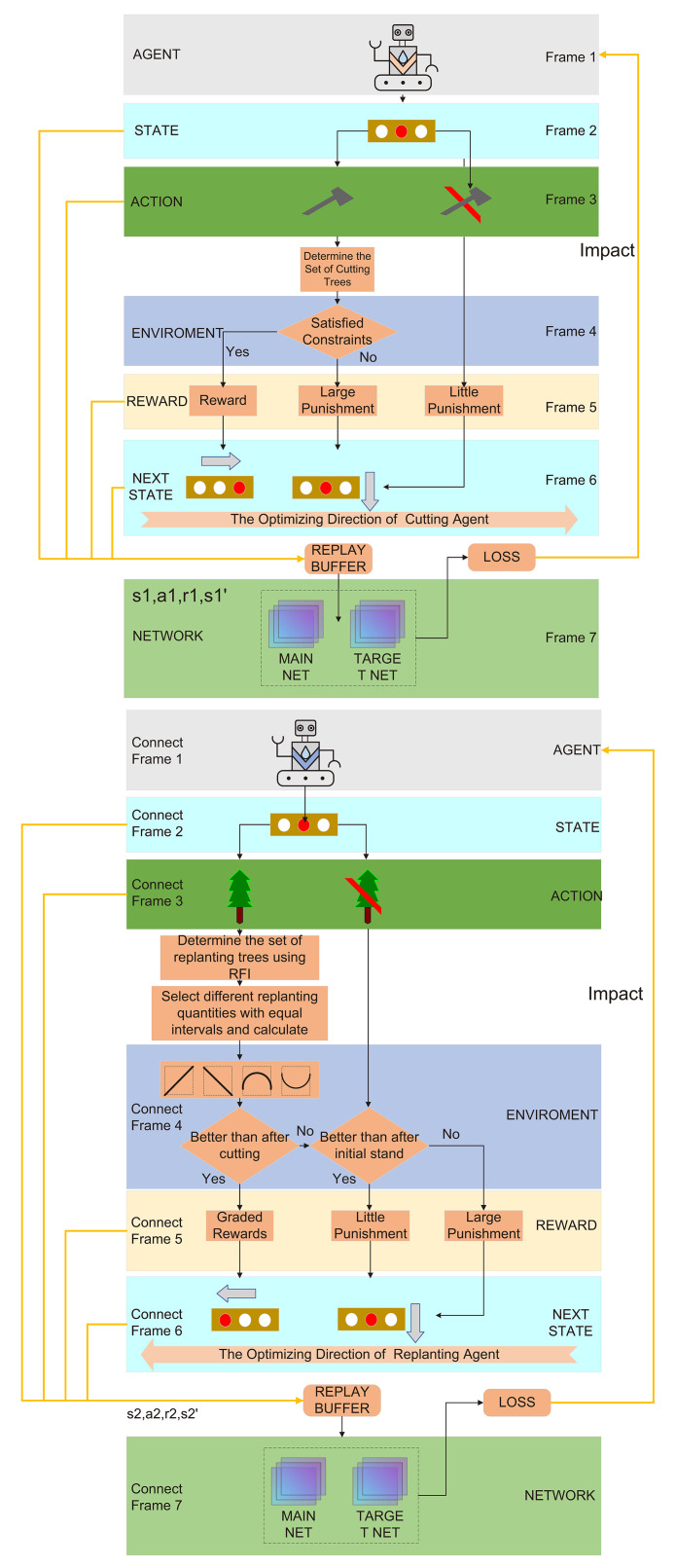
MADQN for stand structure optimization.

The process of solving stand structure optimization using multi-agent deep reinforcement learning is illustrated in **Figure 6** (**Algorithms 1**–**3**).

#### 2.9.2 Solution for dynamic stand structure optimization

During the subsequent optimization process, the selective harvesting and replanting agents continuously collaborate and interact, iteratively optimizing the current stand condition to achieve the most optimal stand structure. In each optimization cycle, the agents learn from environmental feedback, gradually approaching the structural characteristics of an ideal stand. However, real-world stand structures are influenced by multiple factors, and relying solely on selective harvesting and replanting may not directly achieve the desired stand structure. Therefore, a stand structure prediction model is incorporated as a crucial step to further enhance the accuracy and effectiveness of the optimization process.

The stand structure prediction model is used to forecast tree growth trends over a future period based on the current stand condition, as well as the potential changes after selective harvesting and replanting. After providing predictions on the current stand status, the agents further optimize the coordination between harvesting and replanting. At this stage, the selective harvesting and replanting agents not only rely on their original decision framework but also adjust the stand structure based on the prediction results. This process operates as a cyclic feedback mechanism, where each optimization generates a new stand condition, which in turn serves as the starting point for the next round of optimization. After each selective harvesting and replanting step, the updated stand condition is fed into the stand structure prediction model, and the model’s prediction results influence the next round of harvesting and replanting decisions. Through this approach, harvesting and replanting decisions are dynamically adjusted, ensuring that each step moves closer to an ideal stand structure. This iterative optimization process allows the model to identify the best regulatory strategies in a complex and dynamically changing stand environment. By avoiding a fixed strategy from the outset, the optimization process becomes more flexible and adaptive to stand dynamics.

**Figure 7:**
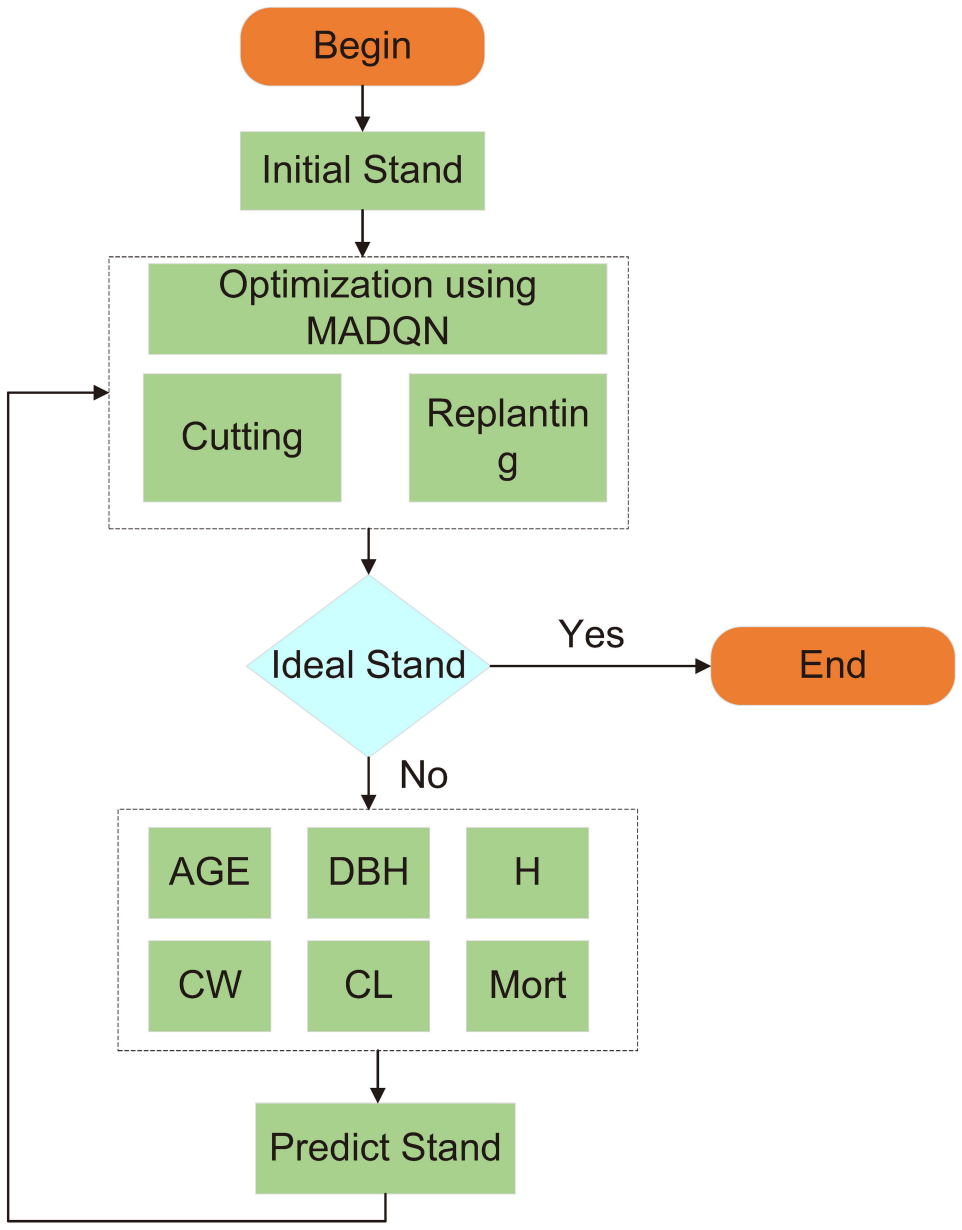
Dynamic optimization process flowchart.

The solution process for dynamic stand structure optimization is illustrated in **Figure 7** (**Algorithm 1**).

#### 2.9.3 Parameter settings

The parameter settings for the solution algorithm used in the experiment are shown in **Table 6**. The initial iteration count and maximum iteration count for the solution algorithm were set to 0 and 1000, respectively. During the optimization process, the stand structure at different time periods is abstracted into a sequence. At the beginning of each iteration, the selective harvesting agent starts at the initial state ( state1=0 ), while the replanting agent starts at the final state ( state2=100 ). Both agents interact with the environment and collaborate with each other to decide whether to move forward (state1=state1+1,state2=state2−1) or move backward (state1=state1−1,state2=state2+1). The iteration ends when the selective redharvesting agent and the replanting agent meet ( state1=state2 ). To compare the performance of MADQN and MAQL, both algorithms were set with the same hyperparameters ( γ=0.9 , lr=0.01 , ϵ=0.9 ). Additionally, for MADQN, the experience replay buffer size was set to 10000, with a batch size of 32. A three-layer fully connected network was used, with each hidden layer containing 24 neurons. These parameter settings were obtained through multiple experiments and fine-tuning to achieve optimal results.

**Table 6:** Parameter settings of the solution algorithm.

Algorithms	Settings	Meaning
MADQN	W=0	Initial iteration
	$W_{max}=10000$	Upper limit of iterations
	state1=0	The agent1’s initial location
	$state1_{max}=50$	The agent1’s permitted farthest move distance
	state2=100	The agent2’s initial location
	$state2_{max}=50$	The agent2’s permitted farthest move distance
	layer=3	Neural network depth
	buffer_size=10000	Replay buffer capacity
	batch_size=32	Batch size for sampling from the replay buffer
	γ=0.9	Discount factor
	lr=0.01	Learning rate
	ϵ=0.9	exploration rate for ϵ -greedy strategy
	a=150,b=−50,c1=100,c2=50,c3=10,c4=1,c5=−1,c6=−50	Reward and punishment values
MAQL	W=0	Initial iteration
	$W_{max}=10000$	Upper limit of iterations
	state1=0	The agent1’s initial location
	$state1_{max}=50$	The agent1’s permitted farthest move distance
	state2=100	The agent2’s initial location
	$state2_{max}=50$	The agent2’s permitted farthest move distance
	γ=0.9	Discount factor
	lr=0.01	Learning rate
	ϵ=0.9	exploration rate for ϵ -greedy strategy
	a=150,b=−50,c1=100,c2=50,c3=10,c4=1,c5=−1,c6=−50	Reward and punishment values

In structured forest management, neighborhood comparison, representing size differentiation and competition intensity, complete mingling, indicating species segregation, and uniform angle index, describing horizontal distribution patterns, are the three most important spatial structure indexes. Considering the limitations of *Pinus yunnanensis* secondary forest plots, (U≤0.5,Mc≥0.75,0.475≤W≤0.517 ) is selected as the ideal stand structure characteristic for dynamic stand structure optimization (Gangying et al., 2005, 2018).

## 3 Results

To verify the effectiveness of the multi-agent deep reinforcement learning solution in stand structure optimization, this study selected five standard plots with different densities and site conditions for simulation experiments. For optimizing the current stand condition, a comparative experiment was conducted between the MADQN and MAQL under the same selective harvesting and replanting methods to evaluate the optimization advantages of MADQN. In the dynamic optimization process, MADQN was integrated with stand structure prediction to enable dynamic adjustments and optimization of the stand structure over time.

### 3.1 Results of simulated harvesting optimization

#### 3.1.1 Current stand structure optimization

**Figure 8:**
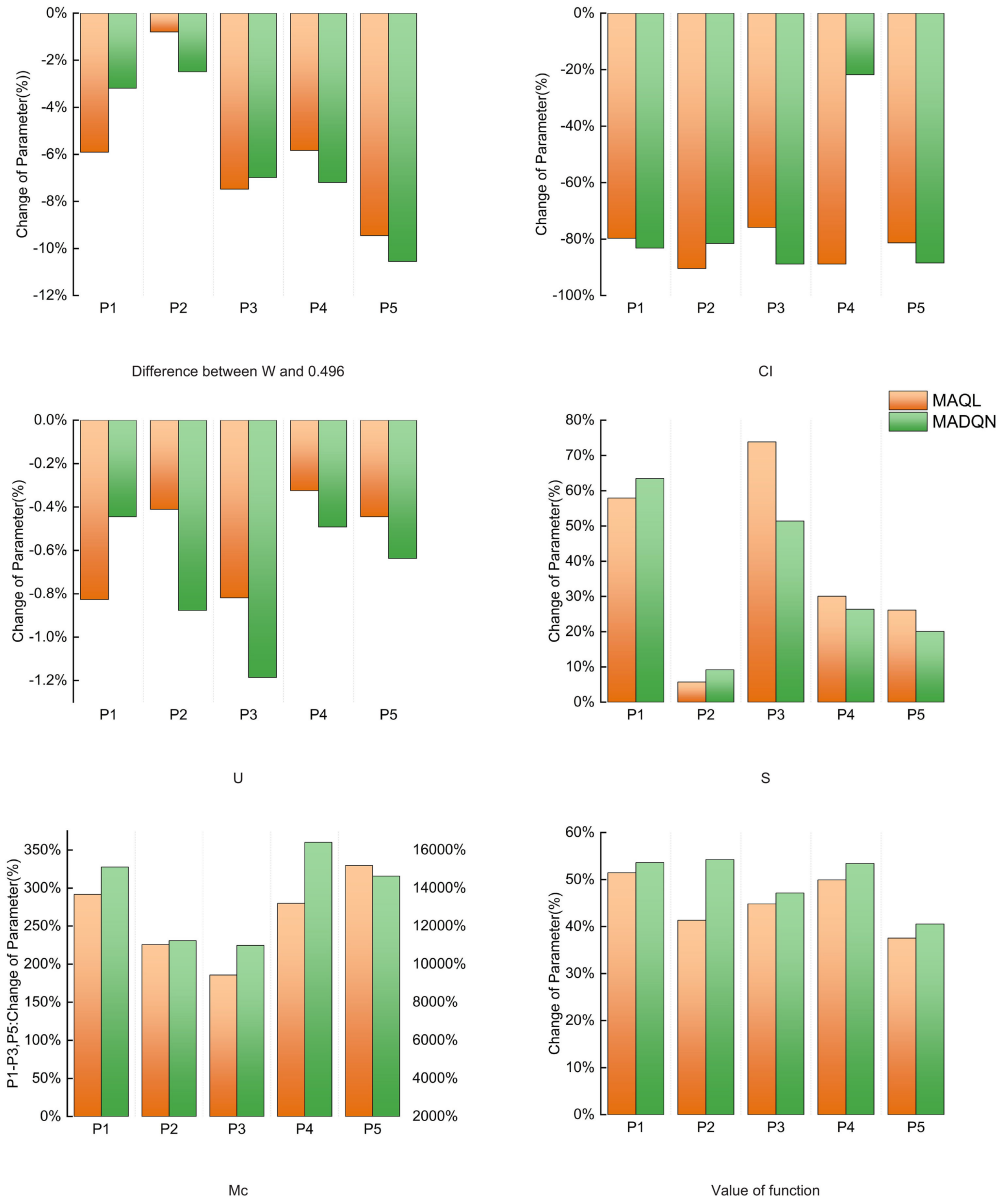
Changes in structure indexes after current stand optimization.

As shown in **Figure 8**, after implementing the two optimization schemes for coordinated selective harvesting and replanting, the stand structure indexes in each plot improved to varying degrees while meeting the constraint conditions, effectively enhancing stand structure. The average uniform angle index in each plot slightly decreased its deviation from 0.496, indicating that the horizontal distribution pattern of the stands became more randomly distributed. The complete mingling index significantly increased across all plots, particularly because the initial mingling degree in each plot was extremely low, leaving ample room for improvement. Notably, in Plot P4, the increase reached 16602.09%. Additionally, the crown competition index decreased substantially in all plots, indicating that tree competition pressure was alleviated after optimization. The stratification index showed a moderate increase, suggesting an improvement in vertical structural complexity and a more diverse vertical distribution pattern. However, the neighborhood comparison showed minimal changes across all plots. This is likely due to the fact that the initial average neighborhood comparison values were already in a moderate growth state, limiting the potential for significant improvement.

As shown in **Table 7**, in the current stand structure optimization, both MADQN and MAQL significantly improved the objective function values. However, in terms of overall improvement, MADQN consistently outperformed MAQL. The objective function values for plots P1 to P5 under MADQN optimization increased from 0.3501, 0.3799, 0.3982, 0.3344, and 0.4294 to 0.5378, 0.5861, 0.5860, 0.5130, and 0.6034, respectively—higher than the values achieved by MAQL (0.5302, 0.5369, 0.5766, 0.5014, and 0.5906). The improvement rate under MADQN reached 49.40%, exceeding the 44.58% achieved by MAQL. These results indicate that MADQN is more effective in optimizing stand structure, guiding it toward a more optimal target state.

**Table 7:** Current stand structure optimization under different optimization schemes across various plots.

Stand Condition	P1	P2	P3	P4	P5	Average	Increase
Initial Stand	0.3501	0.3799	0.3982	0.3344	0.4294	0.3784	
MADQN	0.5378	0.5861	0.5860	0.5130	0.6034	0.5653	49.40%
MAQL	0.5302	0.5369	0.5766	0.5014	0.5906	0.5471	44.58%

**Figure 9:**
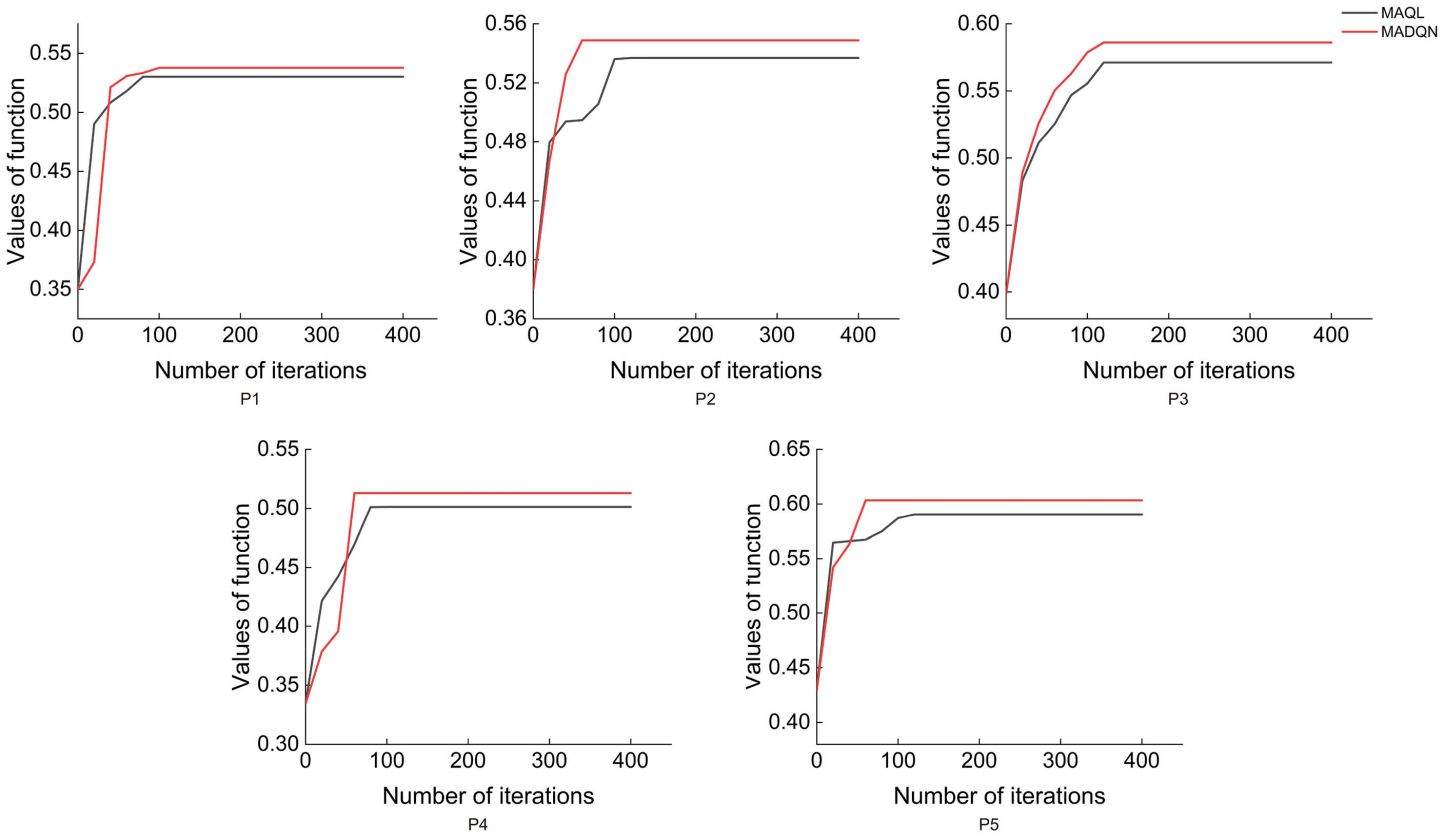
Convergence states of different optimised strategies in different plots.

**Figure 10:**
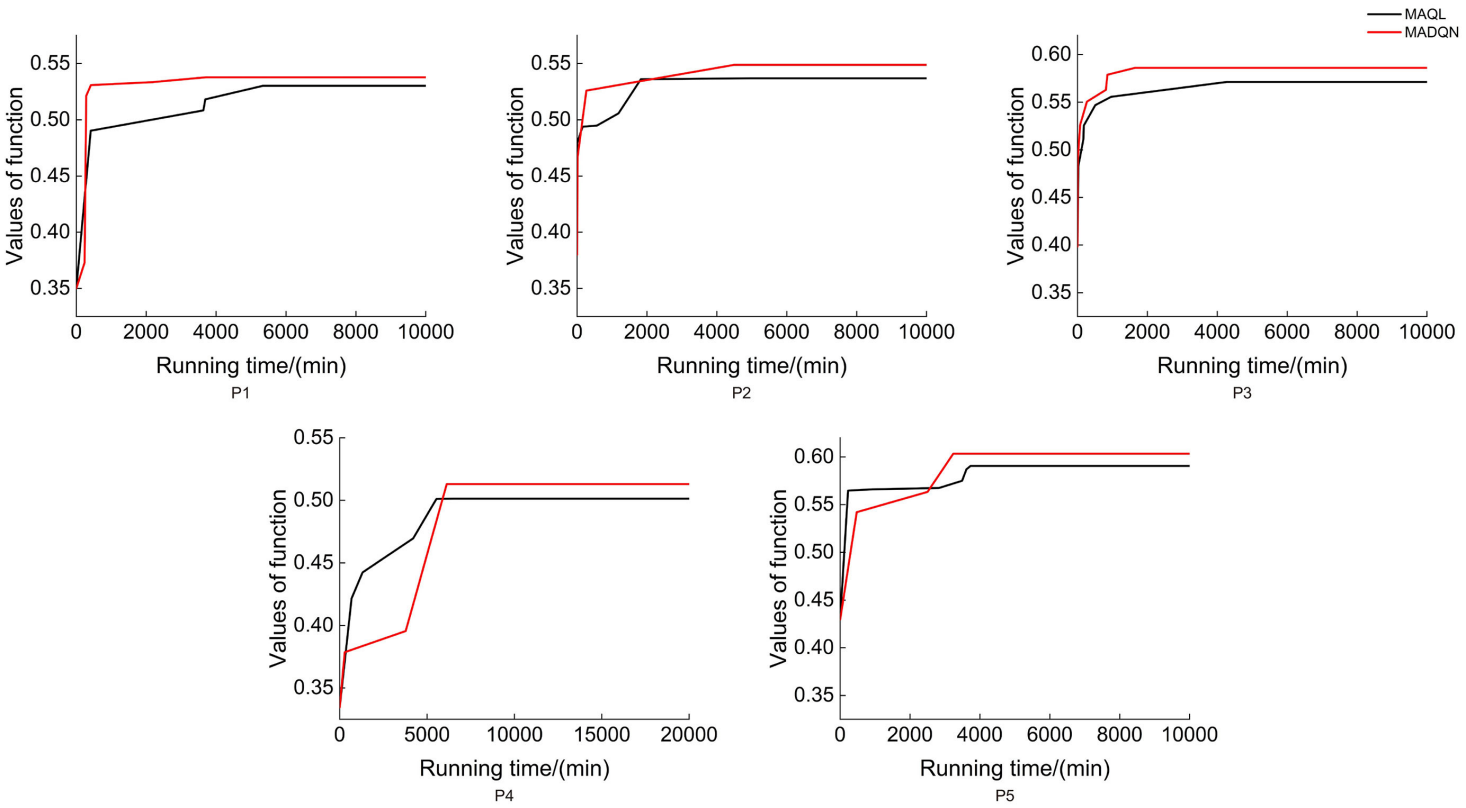
Running time of different optimised strategies in different plots.

As shown in **Figure 9**, MADQN outperformed MAQL in terms of the number of iterations required for optimization. After different numbers of iterations, MADQN exhibited a faster increase in objective function values, especially in Plots P2 and P4. This indicates that MADQN, which utilizes deep neural networks to approximate the Q-function, can find optimal strategies more quickly during the optimization process compared to MAQL, which relies on table-based Q-learning. As a result, MADQN requires less extensive exploration and achieves higher learning efficiency. It is worth noting that in Plots P1 and P3, although MADQN achieved a higher objective function value, MAQL required slightly fewer iterations to converge. This suggests that stand density and site conditions influence convergence performance, and in certain cases, MAQL’s convergence efficiency is not necessarily inferior to MADQN. As shown in **Figure 10**, MADQN also demonstrated better overall performance in terms of computational efficiency. Across different plots, MADQN consistently maintained a lower or comparable runtime curve compared to MAQL, indicating superior time efficiency.

#### 3.1.2 Dynamic stand structure optimization

In the dynamic stand structure optimization of five plots using multi-agent deep reinforcement learning combined with structure prediction, most stand structure indexes showed significant improvements after optimization. The uniform angle index for all plots fell within the ideal range of [0.475, 0.517], indicating that the horizontal distribution pattern had reached a random distribution state. The complete mingling index increased substantially across all plots, shifting from a very low mingling state to a high mingling state. This demonstrates that dynamic optimization not only adjusts the spatial relationships among trees but also enhances stand stability and biodiversity at the species level. Notably, in Plot P4, the mingling index increased from 0.0028 to 0.7505, highlighting the strong adaptability of multi-agent deep reinforcement learning in adjusting tree species composition. Additionally, the crown competition index significantly decreased across all plots, indicating a considerable reduction in competition pressure. The stratification index also improved effectively, enhancing the vertical distribution pattern. In contrast, the neighborhood comparison showed minimal decline across all plots, suggesting that the pre-optimization stand already exhibited a relatively stable size differentiation state. As a result, despite some adjustments during optimization, fluctuations in this index remained small.

As shown in **Table 8**, after incorporating structure prediction for dynamic optimization, the objective function values for all plots experienced significant improvements. Notably, in Plot P4, the objective function value increased from 0.3344 to 0.5863, achieving a remarkable 75.33% increase. Even in Plot P5, where the improvement was relatively smaller, the increase still reached 44.62%. These results indicate that dynamic optimization using multi-agent deep reinforcement learning combined with structure prediction effectively enhances stand structure stability and balance, making it more aligned with an ideal management state.

**Table 8:** Dynamic stand structure optimization under different optimization schemes across various plots.

Sample Plot	Stand Condition	*W*	*Mc*	*CI*	*S*	*U*	*L*	Years	Increase
P1	Initial Stand After Optimizing	0.3630 0.4785	0.1459 0.7615	2.713 0.2041	0.2205 0.3126	0.4960 0.4914	0.3501 0.5718	20	63.32%
P2	Initial Stand After Optimizing	0.5612 0.4869	0.1693 0.7774	3.4130 0.2226	0.2772 0.3450	0.4906 0.4860	0.3799 0.6101	20	60.59%
P3	Initial Stand After Optimizing	0.3867 0.4768	0.1990 0.7786	4.0990 0.2745	0.3286 0.3535	0.4965 0.4852	0.3982 0.6455	35	62.10%
P4	Initial Stand After Optimizing	0.2103 0.4826	0.0028 0.7505	3.1363 0.2475	0.2846 0.3512	0.4930 0.4788	0.3344 0.5863	40	75.33%
P5	Initial Stand After Optimizing	0.5551 0.5102	0.1443 0.7511	2.8202 0.2468	0.2882 0.3573	0.4950 0.4954	0.4294 0.6210	15	44.62%

## 4 Discussion

To avoid the limitations of relying solely on selective harvesting for optimization, this study proposed a multi-objective stand structure optimization scheme based on multi-agent deep reinforcement learning. A simulation experiment was conducted using sample plot data from Pinus yunnanensis secondary forests in Southwest China, where MADQN was applied for the simulation and compared with a multi-agent reinforcement learning optimization scheme. The results showed that under both optimization algorithms, stand structure indexes improved to varying degrees across all plots. However, compared to MAQL, MADQN consistently achieved higher optimization gains across different stand conditions, demonstrating greater adaptability and stability. These findings indicate that multi-agent deep reinforcement learning can learn more optimal strategies in complex environments and achieve more comprehensive optimization in a shorter time.

Traditional multi-agent reinforcement learning is limited by the dimensionality of the state-action space, especially in complex optimization environments like stand structure optimization. Stand structure features exhibit nonlinearity and continuity, making it difficult for MARL to store Q-values in a tabular format. This leads to high storage overhead and low generalization ability. In contrast, multi-agent deep reinforcement learning stores Q-values in a parametric form using neural networks to extract stand structure features. It also employs strategies such as experience replay and target networks to improve training efficiency and stability (Gronauer and Diepold, 2022) (Shen et al., 2022). This allows the same agents, under selective harvesting and replanting measures, to learn more smoothly and approach a globally optimal optimization strategy, with superior convergence capabilities.

Although multi-agent deep reinforcement learning has significant advantages in optimization efficiency and result accuracy for stand structure optimization, the optimal strategy derived from multi-agent deep reinforcement learning is based solely on the current stand structure. However, stand structure is a dynamic system that changes over time due to factors such as tree growth, mortality, and human intervention. Relying solely on optimizing the current stand condition may not meet the long-term management needs of the forest. Currently, stand structure prediction models can utilize tree factors from the current state, such as diameter at breast height, tree height, crown width, crown length, etc., to predict the future trend of these factors, thereby simulating the natural evolution of the stand (Ali, 2019). In this study, multi-agent deep reinforcement learning was combined with structure prediction, providing dynamic environmental information to the optimization process. This allows the optimization strategy to not only apply to the current stand state but also be dynamically optimized based on the predicted stand evolution trend, enabling the agents to formulate more robust strategies while considering long-term dynamic changes. In the combined optimization process with stand structure prediction, the agents can adjust the spatial configuration of trees in advance, based on the predicted stand evolution information, and allocate growth resources more effectively. This ensures that the optimization effect remains stable over the long term. Since changes in stand structure occur gradually, the optimization strategy can dynamically respond to potential future risks, such as intensified competition or mortality, ensuring that the stand structure remains balanced during the succession process and avoiding structural imbalance issues caused by short-term optimization. This approach is better suited to the developmental needs of the stand over different time scales. On the other hand, integrating stand structure prediction also affects the optimization time and strategy adjustment approach. Without prediction, the agents typically require more iterations to adapt to environmental changes. However, by incorporating the prediction model, the agents can obtain future potential structural changes earlier, reducing unnecessary exploration and improving optimization efficiency. Moreover, the long-term trend information provided by the prediction model allows for more precise optimization strategies, preventing fluctuations in stand structure caused by short-term optimization.

From the optimization results, it can be observed that the key stand structure indicators for all plots significantly improved after optimization, and the stand structure approached the ideal stand condition. This validates the feasibility and effectiveness of the method in long-term forest management, indicating that, when considering dynamic stand changes, the stand structure can maintain a reasonable spatial configuration over extended time scales. Compared to the significant changes in other stand structure indicators, the change in neighborhood comparison was relatively small. On one hand, the degree of size differentiation in the plots was already in a stable growth state before optimization (Xuan et al., 2023). On the other hand, multi-agent deep reinforcement learning primarily relies on selective harvesting and replanting as the main regulatory measures. Therefore, the focus of the optimization was on adjusting aspects such as mingling degree, competition pressure, and distribution pattern, while the direct impact on neighborhood comparison was relatively small. Additionally, the optimization time varied across plots. Plot P4 required a longer optimization time, while Plot P5 took relatively less time. This may be related to the initial stand conditions and the difficulty of optimization. Plot P5 had a more balanced initial stand, with its horizontal distribution pattern already close to the ideal stand distribution. The competition pressure and mingling degree were also higher than in Plot P4, allowing for a quicker convergence to an optimal adjustment strategy, resulting in a shorter optimization time. In contrast, Plot P4 had poor mingling, relatively high competition pressure, and a less favorable horizontal distribution pattern. As a result, the optimization process required more rounds of exploration and adjustment to ensure an optimal outcome. Furthermore, the length of the optimization time may also be influenced by the multi-agent deep reinforcement learning algorithm itself. Different exploration strategies and parameter settings directly affect the optimization efficiency. This conclusion is consistent with that obtained by using multi-agent reinforcement learning to solve forest stand structure optimization (Xuan et al., 2024).

Surely, when introducing the deep reinforcement learning algorithm, this research still has the following limitations and aspects that require further refinement: (1) This study only utilized the basic MADQN algorithm within multi-agent deep reinforcement learning. Further research is needed to explore whether other more advanced algorithms and corresponding improvements could be more effective in solving multi-objective stand structure optimization problems. (2) Due to the limited data coverage of the research plots, some of the tree factor predictions still exhibit inaccuracies. Additionally, the current prediction models have certain limitations in addressing the growth variability of individual trees and complex environmental factors. Therefore, more suitable prediction methods should be selected in the future to improve the accuracy of structure prediction. (3) The current dynamic stand structure optimization primarily focuses on adjusting mingling degree, spatial distribution pattern, and competition pressure, with relatively limited optimization of neighborhood comparison. Future optimization strategies will pay more attention to controlling neighborhood comparison to more precisely optimize the diameter structure of the stand, enhancing overall balance and growth stability. (4) In the spatial configuration of replanting trees, the study currently uses a proportional method to select multiple native species. However, due to the limited number of certain species, the basic model is used to determine tree height, crown width, and other tree factors. Future research will further optimize the spatial configuration strategy for replanting trees, making the species composition adjustment more scientific and rational. Additionally, more accurate models will be introduced to improve the prediction accuracy of fundamental tree factors, thus enhancing the adaptability and long-term stability of the optimization scheme.

## 5 Conclusion

This study applies multi-agent deep reinforcement learning to the field of stand structure optimization. The objective function is established using complete mingling, uniform angle index, neighborhood comparison, stratification index, and crown competition index, with selective harvesting and replanting measures for coordinated optimization. Comparative simulation experiments with multi-agent reinforcement learning across different plots showed that the objective function values of multi-agent deep reinforcement learning in each plot were 0.5378, 0.5861, 0.5860, 0.5130, and 0.6034, all higher than those of multi-agent reinforcement learning, which were 0.5302, 0.5369, 0.5766, 0.5014, and 0.5906. These results demonstrate the superiority of multi-agent deep reinforcement learning in stand structure optimization. Considering the dynamic nature of stand structure, combining structural prediction with multi-agent deep reinforcement learning enabled the stand structure in each plot to approach the ideal stand structure within 15–40 years, achieving dynamic optimization of stand structure. This approach provides a scientific basis and decision support for the dynamic optimization of stand structure and has broad application prospects.

## Data Availability

The dataset generated and analyzed during this study is not publicly available due to its ongoing use in current research but is available from the corresponding author, Jianming Wang (wangjianming618@163.com), upon reasonable request.

## Appendix 1

To address stand structure optimization, this study proposes a multi-agent deep reinforcement learning algorithm. The detailed pseudocode is provided below. Due to its length, the decision-making processes of Agent1 and Agent2 in the MADQN framework are presented separately.

Algorithm 1 Overall Process of MADQN for Stand Structure Optimization.

Algorithm 2 Agent 1: Selective harvesting Process.

Algorithm 3 Agent 2: Replanting Process.

### APPENDIX.2

Furthermore, to realize dynamic optimization of stand structure, this study integrates multi-agent deep reinforcement learning for stand structure optimization with structure prediction, and presents the corresponding pseudocode.

Algorithm 4 Stand Structure Dynamic Optimization.
